# Quality of Life and Mood in Relation to Free T3, Free T4 and the FT3/FT4 Ratio in Levothyroxine-Treated Hypothyroidism: A Cross-Sectional Study

**DOI:** 10.3390/medicina62102017

**Published:** 2026-10-18

**Authors:** Gülşah Altun, Enver Çiftel

**Affiliations:** 1 Department of Internal Medicine, Sivas Numune Hospital, 58060 Sivas, Türkiye; 2 Department of Endocrinology and Metabolism, Sivas Numune Hospital, 58060 Sivas, Türkiye; enver-ciftel@hotmail.com

**Keywords:** hypothyroidism, levothyroxine, free T3, FT3/FT4 ratio, quality of life, ThyPRO, Hospital Anxiety and Depression Scale, Hashimoto thyroiditis, thyroidectomy

## Abstract

*Background and Objectives:* Many levothyroxine (LT4)-treated hypothyroid patients report persistent symptoms. We examined thyroid-related quality of life (QoL) and anxiety and depression symptoms in LT4-treated patients with Hashimoto thyroiditis or after thyroidectomy, and their associations with free triiodothyronine (FT3), free thyroxine (FT4), the FT3/FT4 ratio and thyrotropin (TSH). *Materials and Methods:* In this single-centre cross-sectional study, 219 consecutive adults (63 clinic comparison participants without known thyroid disease, 110 LT4-treated Hashimoto patients and 46 LT4-treated post-thyroidectomy patients) completed a Turkish ThyPRO short-form version (nine scales plus an overall QoL-impact item) and the Hospital Anxiety and Depression Scale (HADS). Fasting blood was drawn before the daily LT4 dose. Linear models were adjusted for diagnostic group, age, sex, body-mass index and TSH, with ratio × group interaction testing and group-specific slopes. *Results:* TSH, FT3 and FT4 were all within the reference range in 79% of the comparison group, 57% of Hashimoto and 24% of thyroidectomy patients. ThyPRO and HADS scores differed across groups (all *p* < 0.001). In the primary group-adjusted analysis, each one-unit lower FT3/FT4 ratio was associated with a higher ThyPRO composite (β = −8.6, 95% CI −9.1 to −8.1), HADS-depression (β = −1.4, −1.7 to −1.0) and HADS-anxiety score (β = −1.7, −2.0 to −1.3; all Holm-adjusted *p* < 0.001). Slopes differed between groups for ThyPRO and HADS-anxiety, but point estimates were negative in every group, and associations persisted among participants with all three tests in range (*n* = 124) and after excluding comparison participants with high FT4. TSH was not associated with the outcomes. *Conclusions:* Lower FT3 and FT3/FT4 ratio, but not TSH, were associated with worse thyroid-related QoL and more anxiety and depression symptoms; the association with the ratio persisted in participants with all thyroid tests in range. These cross-sectional associations require confirmation in independent prospective cohorts.

## 1. Introduction

Hypothyroidism is among the most prevalent endocrine disorders, and levothyroxine (LT4) monotherapy is its standard treatment, based on the assumption that peripheral conversion of the pro-hormone thyroxine (T4) to the biologically active triiodothyronine (T3) meets the overall tissue requirement for thyroid hormone [1]. Serum thyrotropin (TSH) within the reference range is used almost universally as the therapeutic target and as a surrogate for restored euthyroidism.

Yet 5–10% of treated patients report persistent fatigue, cognitive complaints and low mood despite a normal TSH, and large patient surveys document prominent dissatisfaction with LT4 therapy [2,3,4]. These residual complaints are only weakly explained by TSH, and the relationship between thyroid function and psychological well-being differs in treated versus untreated individuals [3,5,6]. The reasons remain contested and constitute what has been termed the “enigma of persistent symptoms” [4].

One mechanistic candidate is a subtle failure to restore tissue T3 availability. Because LT4 raises circulating FT4 and secondarily down-regulates type-2 deiodinase activity, LT4-treated patients tend to display higher FT4, with FT3 lower or unchanged, than untreated individuals at a similar TSH, and hence a reduced FT3/FT4 ratio [7,8,9,10]. Athyreotic patients (e.g., after total thyroidectomy) represent the extreme of this spectrum: lacking endogenous thyroidal T3 secretion, they depend entirely on peripheral deiodination, and in one large series about 15% had FT3 below the reference range despite a normal TSH [7]. Serum FT3 itself, and the FT3/FT4 ratio, have therefore been examined as peripheral markers of the thyroid-hormone profile achieved on LT4 [7,10]; whether either reliably indexes tissue-level thyroid status, however, remains uncertain and is not established by these studies.

Whether this biochemical pattern relates to patient-reported outcomes in different causes of LT4-treated hypothyroidism has rarely been examined in one cohort; the biochemical pattern of LT4 therapy is well described [7,10], but whether it maps onto how patients feel is unclear. We hypothesised that FT3 and the FT3/FT4 ratio would be more closely associated than TSH with health-related quality of life (HRQoL) and with anxiety and depression symptoms. We tested this by comparing thyroid-specific HRQoL (ThyPRO) and anxiety and depression symptoms (HADS) among controls without known thyroid disease, LT4-treated patients with Hashimoto thyroiditis and LT4-treated patients after thyroidectomy for benign goitre, and by estimating the associations of FT3 and the FT3/FT4 ratio with these outcomes after adjustment for diagnostic group. Hashimoto thyroiditis and post-thyroidectomy hypothyroidism were regarded as distinct clinical conditions, not as ordered stages of one disease process.

## 2. Materials and Methods

### 2.1. Study Design and Participants

This single-centre, cross-sectional study was conducted at the endocrinology and internal-medicine outpatient clinics of Sivas Numune Hospital, Sivas, Türkiye, between 15 January and 15 July 2026, after ethics approval. All consecutive eligible adults attending during this period were invited; of 239 invited individuals, 20 declined and 219 were enrolled (Figure S1). Three groups were defined: (i) a clinic comparison group (*n* = 63; hereafter “controls”), who attended the same clinics for non-thyroid reasons and had no known thyroid disease or thyroid medication; because thyroid autoantibodies were not measured, this group is not an autoimmunity-free healthy reference population; (ii) patients with Hashimoto thyroiditis (*n* = 110), diagnosed by positive thyroid autoantibodies and/or characteristic ultrasonography, all receiving LT4 replacement; and (iii) patients with primary hypothyroidism following subtotal thyroidectomy performed for benign thyroid disease (*n* = 46), all on stable LT4 replacement. The indications for surgery were multinodular goitre or large compressive goitre; no patient had Graves’ disease or had undergone surgery for malignancy, and none was receiving combined LT4 + liothyronine therapy. All surgical patients were at least 12 months post-operative at assessment. All patients were on a stable LT4 dose; individual doses were not available as an analysable variable. A TSH within the reference range was not an inclusion criterion; thyroid-function status at assessment is reported in Table 1. Thyroid autoantibodies were not measured in all participants, including controls, and were therefore not included as a study variable. The comparison group comprised adults attending the same clinics during the same period for reasons unrelated to the thyroid; they were recruited consecutively under the same eligibility and exclusion criteria as the patients, had no history of thyroid disease, thyroid surgery or thyroid medication, and underwent the same fasting blood sampling and questionnaire administration. In this group, TSH was within the reference range in 62 of 63 participants and FT4 was above the reference range in 13; these participants were retained in the primary analysis and excluded in a sensitivity analysis (Section 3.4).

Inclusion required age ≥ 18 years and the ability to complete the questionnaires. Exclusion criteria were pregnancy or lactation; a known psychiatric disorder or current use of antidepressant, anxiolytic or antipsychotic medication; active malignancy, including thyroid cancer; decompensated cardiac, hepatic or renal disease; use of agents known to interfere with thyroid function or its measurement (amiodarone, lithium, systemic glucocorticoids, high-dose biotin); and any change in LT4 dose within the preceding three months. The study was approved by the Sivas Cumhuriyet University Clinical Research Ethics Committee (decision no. 2025-12/27, dated 11 December 2025) and conducted in accordance with the Declaration of Helsinki (2024 revision). All participants gave written informed consent.

### 2.2. Laboratory and Clinical Measurements

Under a uniform protocol, venous blood was drawn in the morning after an overnight fast and, in patients, before the daily LT4 dose. Serum TSH, FT4 and FT3 were measured in the institutional clinical laboratory by electrochemiluminescence immunoassay on a Roche cobas 8000 analyser (Roche Diagnostics, Mannheim, Germany) (reference ranges: TSH 0.33–5.33 mIU/L; FT4 0.58–1.38 ng/dL; FT3 2.13–4.50 pg/mL). Assays were run within the laboratory’s routine internal quality-control and external quality-assessment programmes, with analytical-imprecision targets derived from biological variation (desirable coefficient of variation ≤9.7% for TSH, ≤3.0% for FT4 and ≤4.0% for FT3). The FT3/FT4 ratio was calculated for each participant as FT3 (pg/mL) divided by FT4 (ng/dL), in the units reported by the laboratory; because its numerical value depends on the units, multiplying it by 0.119 gives the molar ratio (pmol/L divided by pmol/L). Anthropometry (body-mass index, waist circumference), fasting glucose, lipid profile, uric acid, hepatic and renal indices, haemogram and sex hormones (follicle-stimulating hormone, luteinising hormone, oestradiol, total testosterone and sex-hormone-binding globulin) were recorded from the same sample (Table S1).

### 2.3. Patient-Reported Outcomes

Thyroid-specific HRQoL was assessed with a Turkish translation of the ThyPRO-39 short form of the Thyroid-Related Patient-Reported Outcome questionnaire [11,12,13,14]. The version administered in this study comprised nine scales (hypothyroid symptoms, tiredness, cognitive complaints, anxiety, depressivity, emotional susceptibility, impaired social life, impaired daily life and cosmetic complaints) and the single overall QoL-impact item; the goitre-symptom, hyperthyroid-symptom and eye-symptom scales of the complete ThyPRO-39 were not part of the administered version, so no data exist for them. All scales run 0–100, with higher scores indicating worse HRQoL. A composite score was calculated as the unweighted mean of the nine scale scores and the overall QoL-impact item; because it omits three standard scales, it is not directly comparable with composite scores derived from the complete ThyPRO-39. A Turkish validity and reliability study of the full 84-item ThyPRO has been published [15]; the 39-item short form has not been separately validated in Turkish. Anxiety and depression symptoms were measured with the Hospital Anxiety and Depression Scale (HADS), a 14-item screening instrument with anxiety (HADS-A) and depression (HADS-D) subscales scored 0–21 [16]; it identifies probable cases and does not establish a psychiatric diagnosis. The Turkish HADS has established validity and reliability [17]. The prespecified cut-off for a probable case was ≥8 on either subscale [16]; because the Turkish validation study proposed a cut-off of ≥11 for anxiety (and ≥8 for depression) [17], and ≥11 denotes a definite case on the original scale [16], all prevalence and logistic analyses were repeated at ≥11 for both subscales as a sensitivity analysis added during revision. Questionnaires were administered by investigators and allied health staff who were unaware of the participant’s group allocation.

### 2.4. Statistical Analysis

Age, BMI and waist circumference are summarised as mean ± SD and compared by one-way ANOVA (Kruskal–Wallis tests gave the same conclusions); all other continuous variables are summarised as median (interquartile range, IQR; quartiles by linear interpolation), and the same statistic is used for each variable throughout the text, tables and figures. Categorical variables are given as counts (percentages). Other between-group comparisons used the Kruskal–Wallis test with Dunn post hoc tests (Bonferroni-adjusted) and the eta-squared effect size, η^2^ = (H − k + 1)/(n − k); Kruskal–Wallis *p*-values across the 14 patient-reported outcomes were additionally adjusted by the Benjamini–Hochberg procedure. Thyroid indices were compared between the two patient groups, and between controls and each patient group, with the Mann–Whitney U test, and proportions by the χ^2^ test. Spearman correlations between thyroid indices (TSH, FT3, FT4 and the FT3/FT4 ratio) and outcomes were computed in the whole cohort and within each group.

The primary models were multivariable linear regressions in all participants (*n* = 219), with the FT3/FT4 ratio as the exposure, adjusted for diagnostic group, age, sex, body-mass index (BMI) and TSH. Effect modification by group was tested by adding ratio × group interaction terms (F-test, with covariate effects common to all groups), and group-specific slopes were estimated in separate models within each group, adjusted for age, sex, BMI and TSH. Parallel models used FT3 instead of the ratio. To examine whether the ratio carried information beyond its components, a fixed set of models was compared, each adding to the base model (group, age, sex, BMI and TSH) one of the following: FT3; FT4; FT3 and FT4; the ratio; log FT3 and log FT4; the log ratio; or FT3, FT4 and the ratio. Models were compared by the partial R^2^ of the added terms, the Akaike and Bayesian information criteria (AIC, BIC) and the root-mean-square error (RMSE) of 10-fold cross-validation repeated 10 times, and nested F-tests examined whether the ratio added to FT3, or to FT3 and FT4. Model assumptions were checked with the Shapiro–Wilk test of residuals, the Breusch–Pagan test and variance inflation factors. Stability was assessed with heteroscedasticity-consistent (HC3) standard errors, Huber robust regression, and refits after excluding influential observations (Cook’s distance > 4/n) or the single extreme ratio value (7.14). Sensitivity analyses repeated the primary models in participants with TSH within the reference range, in participants with TSH, FT3 and FT4 all within the reference range, in women only, in patients only (with a Hashimoto-versus-thyroidectomy indicator) and after excluding the 13 controls with FT4 above the reference range.

*Multiplicity*. The primary analysis was defined as the association of the FT3/FT4 ratio with the three main outcomes (ThyPRO composite, HADS-depression and HADS-anxiety) in the group-adjusted model in all participants, and these three *p*-values were adjusted by the Holm method. All other analyses (correlations, group-specific and interaction models, sensitivity and model-comparison analyses, logistic and ROC analyses, ThyPRO scale-level comparisons and sex-hormone analyses) are exploratory; they are reported with unadjusted *p*-values unless stated otherwise and are interpreted as supportive rather than confirmatory.

Because no control met three of the four HADS thresholds (quasi-complete separation), probable depression and anxiety (HADS ≥ 8 and, as a sensitivity analysis, ≥11) were modelled within patients (*n* = 156), adjusted for group, age, sex, BMI and TSH; Firth penalised logistic regression was used because of the limited number of events per variable. Receiver-operating-characteristic (ROC) analysis within patients was exploratory: areas under the curve (AUCs) are given with 1000-sample percentile bootstrap 95% confidence intervals (CIs), and the sensitivity and specificity at the Youden-optimal cut-off were corrected for optimism by bootstrap (1000 resamples), because the cut-off was selected in the same sample. To address confounding by diagnostic group, AUCs were also estimated within each patient group when both outcome classes contained at least 10 participants, and a logistic model containing group alone was compared with one containing group and the ratio (difference in AUC with 1000-sample bootstrap CI, likelihood-ratio test and 10-fold cross-validated AUC repeated 10 times). No a priori sample-size calculation was performed; all consecutive eligible patients seen during the six-month recruitment period were included. With 156 patients, a correlation of |r| ≥ 0.23 could be detected with 80% power at a two-sided α of 0.05. There were no missing data for any analysed variable. Two-sided *p* < 0.05 was considered significant within the framework described below. Analyses were performed in Python 3.11 (Python Software Foundation, Beaverton, OR, USA) using SciPy 1.17, statsmodels 0.15, scikit-learn 1.8, NumPy 2.4 and pandas 3.0; figures were produced with Matplotlib 3.10. The study is reported in accordance with the STROBE statement for cross-sectional studies.

## 3. Results

### 3.1. Participant Characteristics

Participant flow is shown in Figure S1; there were no missing data. Demographic, clinical and thyroid-function characteristics are shown in Table 1, and routine biochemistry and sex hormones in Table S1. Age was similar across groups (overall range 27–56 years); BMI and waist circumference were higher in both patient groups, and the proportion of women differed (*p* < 0.001), being highest in the Hashimoto group. Median FT3 was 3.30 pg/mL in controls, 2.83 pg/mL in Hashimoto and 2.53 pg/mL in thyroidectomy patients (*p* < 0.001), and the median FT3/FT4 ratio was 2.61, 2.40 and 1.81, respectively (*p* < 0.001). FT4 was highest after thyroidectomy (median 1.32 vs. 1.17 ng/dL in Hashimoto and 1.21 ng/dL in controls; *p* = 0.013); FT4 did not differ between Hashimoto patients and controls (*p* = 0.46), so the lower ratio in the Hashimoto group reflected mainly lower FT3. TSH was within the reference range in 62/63 (98.4%) controls, 96/110 (87.3%) Hashimoto and 32/46 (69.6%) thyroidectomy patients, and TSH, FT3 and FT4 were all within their reference ranges in 50 (79.4%), 63 (57.3%) and 11 (23.9%), respectively. Out-of-range values were mainly FT4 above the upper limit (13, 23 and 21 participants, respectively) and FT3 below the lower limit (0, 25 and 15); TSH was above the upper limit in 9 Hashimoto and 9 thyroidectomy patients and below the lower limit in 1, 5 and 5 participants. A substantial proportion of treated patients, particularly after thyroidectomy, was therefore not biochemically euthyroid at assessment.

### 3.2. Quality of Life and Mood Across Groups

All HADS and ThyPRO outcomes differed across the three groups (Table 2; Figure 1; Kruskal–Wallis *p* < 0.001 for all 14 outcomes, all retained after Benjamini–Hochberg correction), with large effect sizes (η^2^ 0.47–0.75; largest for the ThyPRO composite). Scores were lowest in controls, intermediate in Hashimoto patients and highest in thyroidectomy patients, and all three pairwise contrasts were significant for every outcome (Dunn post hoc test). The Hashimoto group had higher scores than controls for HADS-depression (median 7 vs. 2), HADS-anxiety (8 vs. 4) and the ThyPRO composite (28.7 vs. 8.4; all *p* < 0.001). Probable depression (HADS-D ≥ 8) was present in 0%, 46.4% and 89.1% of controls, Hashimoto and thyroidectomy patients, and probable anxiety (HADS-A ≥ 8) in 3.2%, 57.3% and 95.7%, respectively; at the stricter cut-off of ≥11 the corresponding figures were 0%, 12.7% and 50.0% for depression and 0%, 14.5% and 54.3% for anxiety (all χ^2^ *p* < 0.001; Figure 1e; Table S2). Although the two patient groups had similar TSH (median 3.08 vs. 2.98 mIU/L; *p* = 0.85), thyroidectomy patients had lower FT3 (2.53 vs. 2.83 pg/mL; *p* = 0.022), higher FT4 (1.32 vs. 1.17 ng/dL; *p* = 0.004) and a lower FT3/FT4 ratio (1.81 vs. 2.40; *p* < 0.001) (Figure 2).

### 3.3. Associations of Thyroid Indices with Quality of Life and Mood

In the whole cohort, the FT3/FT4 ratio correlated inversely with the ThyPRO composite (ρ = −0.76), HADS-depression (ρ = −0.59) and HADS-anxiety (ρ = −0.63), and FT3 showed a similar pattern (ρ = −0.66, −0.60 and −0.57; all *p* < 0.001), whereas FT4 correlated weakly and positively (ρ = 0.38, 0.22 and 0.26) and TSH was not correlated with these outcomes (ρ = 0.07, *p* = 0.27; ρ = 0.12, *p* = 0.07; ρ = 0.10, *p* = 0.15) (Figure 3a). Because whole-cohort correlations partly reflect differences between groups, correlations were also examined within each group (Figure 3b–d; Table S3). Within groups, the ratio remained inversely correlated with the ThyPRO composite (ρ = −0.83 in controls, −0.91 in Hashimoto and −0.91 in thyroidectomy patients), whereas correlations with HADS scores were weaker and variable (HADS-depression ρ = −0.23, −0.54 and −0.46; HADS-anxiety ρ = −0.19, −0.64 and −0.26). Within controls and Hashimoto patients, TSH showed weak inverse correlations with the ThyPRO composite and some of its scales. In controls, the inverse correlation of the ratio with the ThyPRO composite reflected mainly a positive correlation with FT4 (ρ = 0.65) rather than with FT3 (ρ = −0.18) (Table S3).

In the primary model including all participants and adjusted for diagnostic group, age, sex, BMI and TSH, each one-unit lower FT3/FT4 ratio was associated with an 8.6-point higher ThyPRO composite (β = −8.59, 95% CI −9.13 to −8.05), a 1.4-point higher HADS-depression score (β = −1.38, −1.71 to −1.04) and a 1.7-point higher HADS-anxiety score (β = −1.67, −2.02 to −1.32; all *p* < 0.001) (Table 3; Figure 4). TSH, age, sex and BMI were not associated with any outcome in these models. The ratio × group interaction was significant for the ThyPRO composite (*p* < 0.001) and HADS-anxiety (*p* = 0.013) but not for HADS-depression (*p* = 0.60). In group-specific models, the ratio was inversely associated with the ThyPRO composite in every group (β = −5.93 in controls, −8.67 in Hashimoto and −9.91 in thyroidectomy patients; all *p* < 0.001) and with HADS-depression in every group (β = −0.97, −1.43 and −1.15; *p* = 0.034, <0.001 and 0.020). For HADS-anxiety, the association was clear in the Hashimoto group (β = −1.99, *p* < 0.001) but was not statistically significant in controls (β = −0.88, *p* = 0.12) or thyroidectomy patients (β = −0.95, *p* = 0.075). Models using FT3 instead of the ratio gave concordant results (Table S4). After Holm adjustment for the three primary outcomes, all three associations remained significant (adjusted *p* < 0.001). The interaction and group-specific estimates are exploratory.

Model residuals were approximately normal (Shapiro–Wilk *p* ≥ 0.21), and the variance inflation factor for the ratio was 1.23. Residual variance was heterogeneous for the ThyPRO composite (Breusch–Pagan *p* < 0.001); HC3 standard errors widened its confidence interval only slightly (−9.71 to −7.47). Huber robust estimates (ThyPRO composite β = −9.03; HADS-depression −1.43; HADS-anxiety −1.71) and refits excluding influential observations or the single extreme ratio value of 7.14 gave similar or slightly larger coefficients (Table S4).

Within patients (*n* = 156), a higher FT3/FT4 ratio was associated with lower odds of probable depression (Firth odds ratio per unit 0.32, 95% CI 0.18–0.53 at HADS-D ≥ 8; 0.30, 0.15–0.55 at ≥11) and of probable anxiety (0.19, 0.08–0.37 at HADS-A ≥ 8; 0.30, 0.16–0.52 at ≥11) after adjustment for group, age, sex, BMI and TSH (Table S2). In exploratory ROC analysis within patients, the AUCs of the ratio ranged from 0.77 to 0.81 (Table 4; Figure S2). Within groups, AUCs were 0.76–0.82 in Hashimoto patients; in thyroidectomy patients they were 0.75 (95% CI 0.59–0.89) for HADS-D ≥ 11 and 0.61 (0.43–0.78) for HADS-A ≥ 11, and could not be estimated at the ≥8 cut-offs because only 5 and 2 patients, respectively, were below them (Table S6). A logistic model containing group alone gave AUCs of 0.68–0.71; adding the ratio raised them to 0.82–0.85 (difference 0.12–0.17, all bootstrap 95% CIs excluding zero; likelihood-ratio *p* < 0.001 for all four outcomes), and the cross-validated AUCs were 0.59–0.62 and 0.81–0.84, respectively. After bootstrap correction for optimism, sensitivity and specificity at the Youden-optimal cut-offs (FT3/FT4 1.86–2.25) were 0.63–0.77 and 0.64–0.81, respectively. These cut-offs are specific to the units and assay used here and have not been validated in an independent cohort.

### 3.4. Sensitivity Analyses

The association of the FT3/FT4 ratio with each outcome was consistent across sensitivity analyses (Table S4): among participants with TSH within the reference range (*n* = 190; ThyPRO composite β = −8.98, HADS-depression −1.41, HADS-anxiety −1.75), among those with TSH, FT3 and FT4 all within the reference range (*n* = 124; −7.75, −1.59 and −1.40; all *p* < 0.001), in women only (*n* = 183; −8.71, −1.38 and −1.63), in patients only (*n* = 156; −8.93, −1.41 and −1.79) and after excluding the 13 controls with FT4 above the reference range (*n* = 206; −8.66, −1.43 and −1.69; all *p* < 0.001). Among participants with all three tests in the reference range, median ThyPRO composite scores were 7.4 in controls, 25.9 in Hashimoto and 43.4 in thyroidectomy patients, and median HADS-depression and HADS-anxiety scores were 2.5 and 3.0, 7.0 and 7.0, and 10.0 and 10.0, respectively. Serum total testosterone was not correlated with any outcome in women (all *p* > 0.8); in men (*n* = 36), it showed a weak inverse correlation with HADS-anxiety (ρ = −0.33, *p* = 0.049) but not with HADS-depression (ρ = −0.27, *p* = 0.12) or the ThyPRO composite (ρ = −0.26, *p* = 0.12) (Table S3).

## 4. Discussion

In this single-centre cohort, thyroid-specific quality of life and anxiety and depression symptoms differed markedly between controls, LT4-treated patients with Hashimoto thyroiditis and LT4-treated patients after thyroidectomy for benign goitre: scores were worse in Hashimoto patients than in controls, and worst after thyroidectomy. A lower FT3/FT4 ratio, and lower FT3, were associated with worse scores after adjustment for diagnostic group, age, sex, BMI and TSH. For the ThyPRO composite and HADS-depression the association was present within each group, although its strength differed between groups, and it persisted among participants in whom TSH, FT3 and FT4 were all within the reference range. TSH itself was not associated with the outcomes in the whole cohort or in adjusted models. Because the design is cross-sectional, these relationships are associative and not necessarily causal.

These associations are compatible with known LT4 physiology. As T4 is a pro-hormone requiring deiodination to active T3, euthyroidism on LT4 depends on peripheral deiodinase activity [18]. A normalised TSH is typically achieved only at a high-normal FT4, which restrains type-2 deiodinase and lowers the FT3/FT4 ratio [7,8,18]; classic work in thyroidectomised rats showed that thyroxine alone does not restore T3 in all tissues [19]. Athyreotic patients frequently fail to normalise FT3 despite a normal TSH [7], and serum FT3 itself tracks metabolic markers in such patients [10]. Our surgical group underwent subtotal—not total—thyroidectomy and retains variable residual tissue, yet showed the lowest FT3/FT4 ratio (median 2.61, 2.40 and 1.81 in controls, Hashimoto and thyroidectomy patients), which may reflect reduced functional thyroid mass together with LT4 dependence, although remnant volume was not measured.

Not all of our findings follow the typical LT4 pattern. LT4-treated patients are generally reported to have higher FT4, with FT3 lower or unchanged, than untreated individuals at a similar TSH [7,8,9,20], and this was the case after thyroidectomy. In the Hashimoto group, however, median FT4 did not differ from that of controls (1.17 vs. 1.21 ng/dL; *p* = 0.46), as also reported in patients with Hashimoto thyroiditis on long-term LT4 [21], and the lower ratio was driven by lower FT3. Possible explanations include residual endogenous thyroid function in many Hashimoto patients and the fact that a normal TSH was not required for inclusion: TSH was above the reference range in nine Hashimoto patients, suggesting under-replacement in some. The FT3/FT4 ratio therefore reflects both FT3 and FT4, and it should not be interpreted as a direct measure of tissue thyroid status or of deiodinase activity.

The absence of a TSH–outcome association in the whole cohort and in adjusted models is consistent with prior work: small changes in LT4 dose and TSH did not alter well-being in a randomised trial [22], residual symptoms are only weakly explained by TSH [3,6], and in the HUNT study the relationship between thyroid function and mood differed between thyroxine users and non-users [5]. When FT3 and the FT3/FT4 ratio are examined instead, an inverse association emerges. In exploratory model comparisons, the ratio fitted these data better than FT3 or FT4 alone or the two entered separately, but adding FT3 and FT4 to the ratio further improved the fit for the ThyPRO composite; the ratio is therefore best regarded as a convenient summary of the FT3–FT4 balance rather than a uniquely informative marker. The two patient groups had similar TSH (*p* = 0.85) yet differed in FT3, FT4, the FT3/FT4 ratio and symptom burden, suggesting that TSH alone may not capture all of the variation in how LT4-treated patients feel [2,4]. The weak inverse within-group correlations of TSH with some ThyPRO scales were not reflected in the adjusted models and should be interpreted with caution.

Our results align with disease-specific studies: impaired quality of life is documented across benign thyroid disorders [11], persists in Hashimoto patients on long-term LT4 [21], and is increased by autoimmunity independently of thyroid function [23]; quality of life also improves after total thyroidectomy for benign goitre [24]. The biochemical pattern itself is not novel—higher FT4, lower FT3 and a reduced FT3/FT4 ratio on LT4 are established [7,10]; our contribution is to relate this inexpensive index to patient-reported outcomes both within and across two different causes of LT4-treated hypothyroidism in one cohort. Hashimoto thyroiditis and post-thyroidectomy hypothyroidism are distinct clinical conditions, and the differences between them should not be read as a biological gradient of hypothyroidism.

A genetic mechanism has been proposed: the DIO2 Thr92Ala variant has been linked to baseline well-being and to the response to combined T4 + T3 therapy [25], and reduces deiodinase-2 activity and serum T3 [26]. The evidence is not uniform—in the population-based LifeLines cohort the variant was unrelated to thyroid parameters, quality of life or cognition [20]. Notably, that same cohort found LT4 users to have higher FT4, lower FT3, a lower FT3/FT4 ratio and poorer quality of life than non-users [20], concordant with our data. We did not genotype participants.

The magnitude of the findings requires comment. The prevalence of probable depression (89%) and anxiety (96%) at the HADS cut-off of 8 after thyroidectomy is higher than usually reported, although it fell to 50% and 54% at the stricter cut-off of 11; HADS is a screening instrument, and these figures indicate symptom burden rather than psychiatric diagnoses. The strength of the associations is also unusually high for patient-reported outcomes: the primary model (ratio, group, age, sex, BMI and TSH) explained 96% of the variance of the ThyPRO composite. Questionnaires were administered by staff unaware of group allocation, but participants knew their own diagnosis, and single-centre recruitment, the psychological burden of chronic disease and surgery, and the use of a short form not validated in Turkish may all have contributed. These estimates therefore need replication in independent cohorts. Randomised trials of combined LT4 + LT3 or liothyronine therapy have shown inconsistent benefit [27,28], and guidelines endorse LT4 monotherapy while allowing a trial of combination therapy in selected, persistently symptomatic patients [29,30], an approach also discussed in expert reviews [31], balanced against concerns over liothyronine misuse and limited long-term safety data [32]. Our cross-sectional data do not show that raising FT3 or the FT3/FT4 ratio would improve symptoms and cannot be used to select patients for combination therapy; whether patients with a low ratio respond differently to treatment would need to be tested in prospective or randomised studies.

### Strengths and Limitations

Strengths include the inclusion of two causes of LT4-treated hypothyroidism and a clinic comparison group from the same clinics in one cohort, consecutive recruitment with a low refusal rate (20 of 239) and no missing data, standardised fasting sampling before the LT4 dose, and an analytic strategy that adjusted for diagnostic group, tested effect modification, reported group-specific estimates and examined model stability in several sensitivity analyses.

Several limitations apply. First, the cross-sectional design precludes causal inference; a low FT3/FT4 ratio or FT3 may be a marker rather than a mediator, and reverse causation or shared determinants cannot be excluded. Second, a normal TSH was not required for inclusion, and only 57% of Hashimoto and 24% of thyroidectomy patients had all three thyroid tests within the reference range; the associations persisted in this subgroup, but it included only 11 thyroidectomy patients. Third, the surgical group underwent subtotal—not total—thyroidectomy, so residual thyroid mass varied; thyroid or remnant volume was not quantified by ultrasonography, and in Hashimoto patients the hormone balance may also vary with thyroid volume. Fourth, individual LT4 dose and time since surgery were not available as analysable variables, and thyroid autoantibodies were not measured in all participants, including controls; because thyroid autoimmunity may affect quality of life independently of thyroid function [23], residual confounding by autoimmunity is possible, and some controls may have had undiagnosed thyroid autoimmunity; FT4 was above the reference range in 13 controls, although excluding them did not change the results. Menopausal status was not recorded as a study variable, although FSH and LH were higher and oestradiol lower in women in the patient groups (Table S1). Fifth, thyroid hormones were measured once, by immunoassay in a single laboratory; FT3 and FT4 immunoassays differ between platforms, the FT3/FT4 ratio depends on the units used, and the cut-offs reported here are assay- and unit-specific and require validation in an independent cohort before any clinical use. Sixth, the ThyPRO version administered comprised nine scales and the overall QoL-impact item, without the goitre, hyperthyroid and eye scales; our composite is therefore not directly comparable with the standard ThyPRO-39 composite, and the 39-item short form has not been validated in Turkish, although the full ThyPRO has been [15]. Seventh, HADS identifies probable, not diagnosed, cases. Eighth, only the three primary associations were adjusted for multiplicity; the numerous secondary, sensitivity, model-comparison, logistic, ROC and sex-hormone analyses were not, and are exploratory. Ninth, the comparison group was a clinic-based sample of adults attending for non-thyroid reasons rather than a population-based healthy control group: thyroid autoantibodies were not assessed, FT4 was above the reference range in 13 participants, and their own medical conditions may have influenced quality of life and mood. Although excluding participants with high FT4 did not change the results, this group may not represent the general euthyroid population, and differences between it and the patient groups should be interpreted with caution. Finally, the study was single-centre, with unequal group sizes, a female predominance and no a priori sample-size calculation, which limits generalisability.

## 5. Conclusions

In this cross-sectional cohort of LT4-treated patients with Hashimoto thyroiditis or post-thyroidectomy hypothyroidism, thyroid-related quality of life was impaired and anxiety and depression symptoms were common, including among patients whose thyroid tests were within the reference range. Lower FT3 and a lower FT3/FT4 ratio, but not TSH, were associated with worse outcomes after adjustment for diagnostic group, although the strength of the association differed between groups. Whether these indices lie on a causal pathway or are markers of other factors cannot be determined from these data and requires prospective and interventional studies with assay-specific validation. Meanwhile, symptomatic LT4-treated patients may warrant clinical evaluation even when TSH is within the reference range.

## Figures and Tables

**Figure 1:**
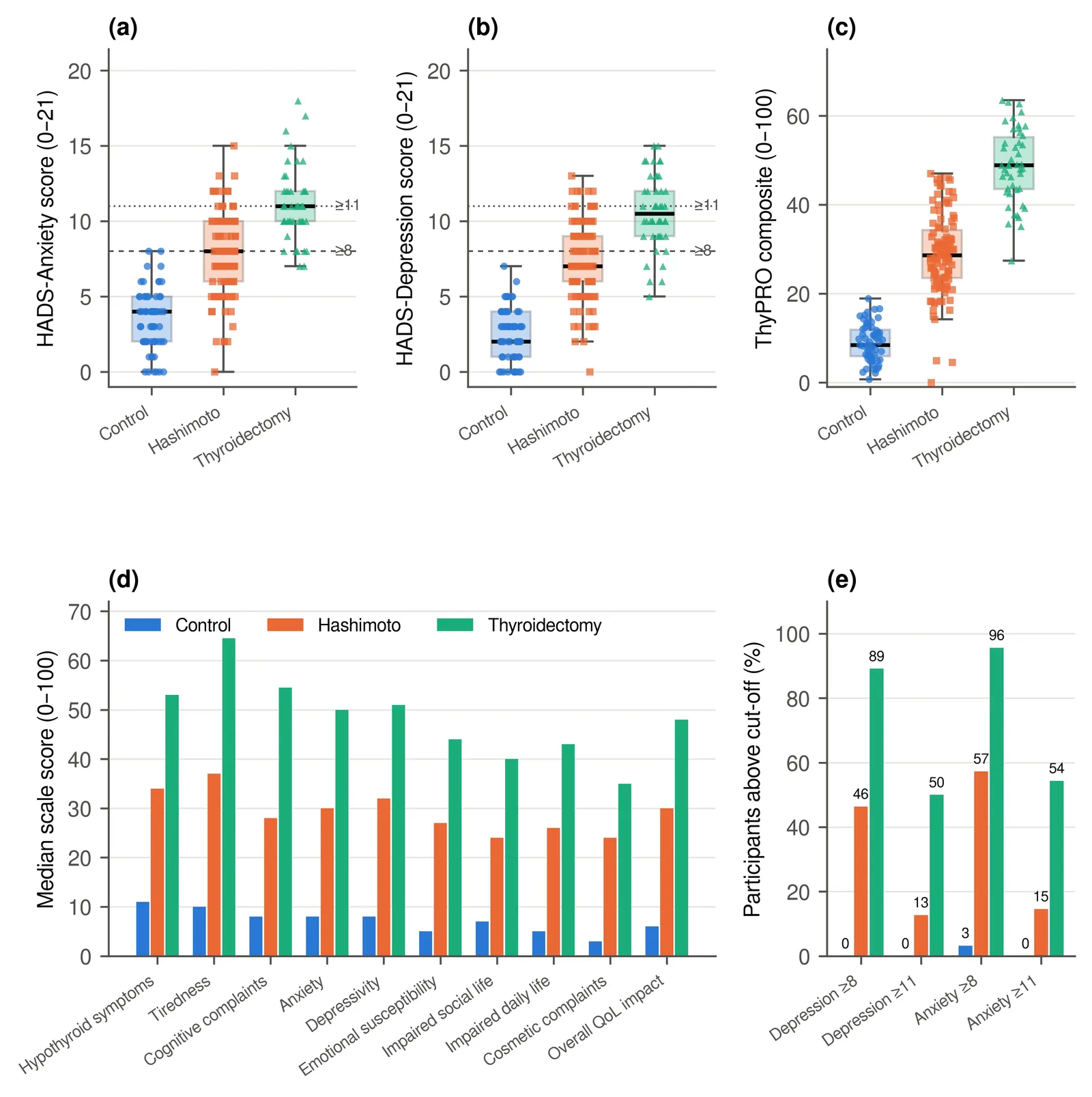
Anxiety and depression symptoms and thyroid-related quality of life by group. (**a**) HADS-anxiety, (**b**) HADS-depression and (**c**) ThyPRO composite scores (boxes, median and IQR; whiskers, 1.5 × IQR; points, individual participants); dashed and dotted lines mark the HADS cut-offs of 8 and 11. (**d**) Median scores of the nine ThyPRO scales and the overall QoL-impact item. (**e**) Percentage of participants with probable depression or anxiety at HADS cut-offs of ≥8 and ≥11. HADS, Hospital Anxiety and Depression Scale; QoL, quality of life.

**Figure 2:**
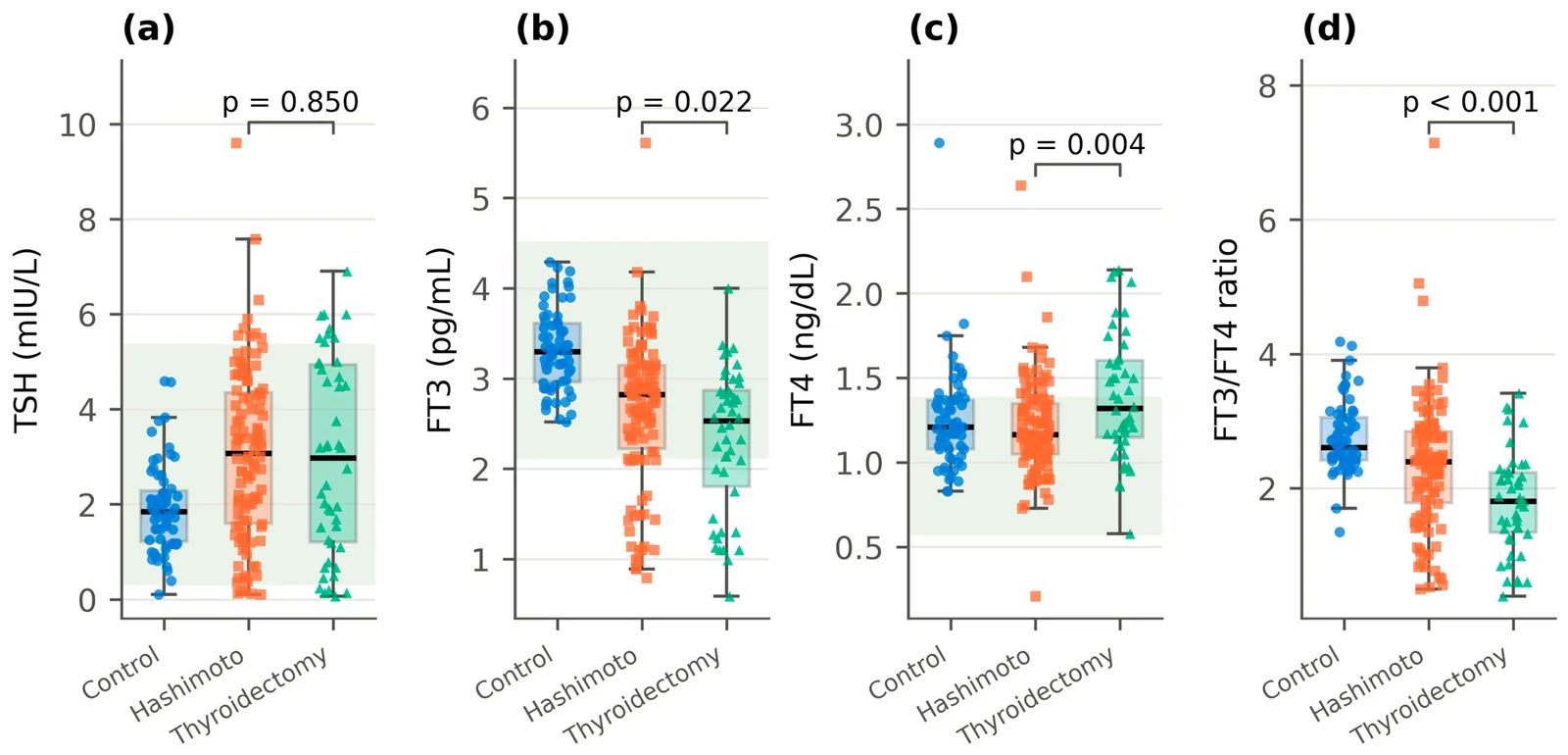
Thyroid-function indices by group: (**a**) TSH, (**b**) FT3, (**c**) FT4 and (**d**) FT3/FT4 ratio (boxes, median and IQR; points, individual participants). Shaded bands show the laboratory reference ranges (no reference range applies to the ratio). Brackets give Mann–Whitney *p*-values for Hashimoto versus thyroidectomy patients, who had similar TSH but differed in FT3, FT4 and the FT3/FT4 ratio.

**Figure 3:**
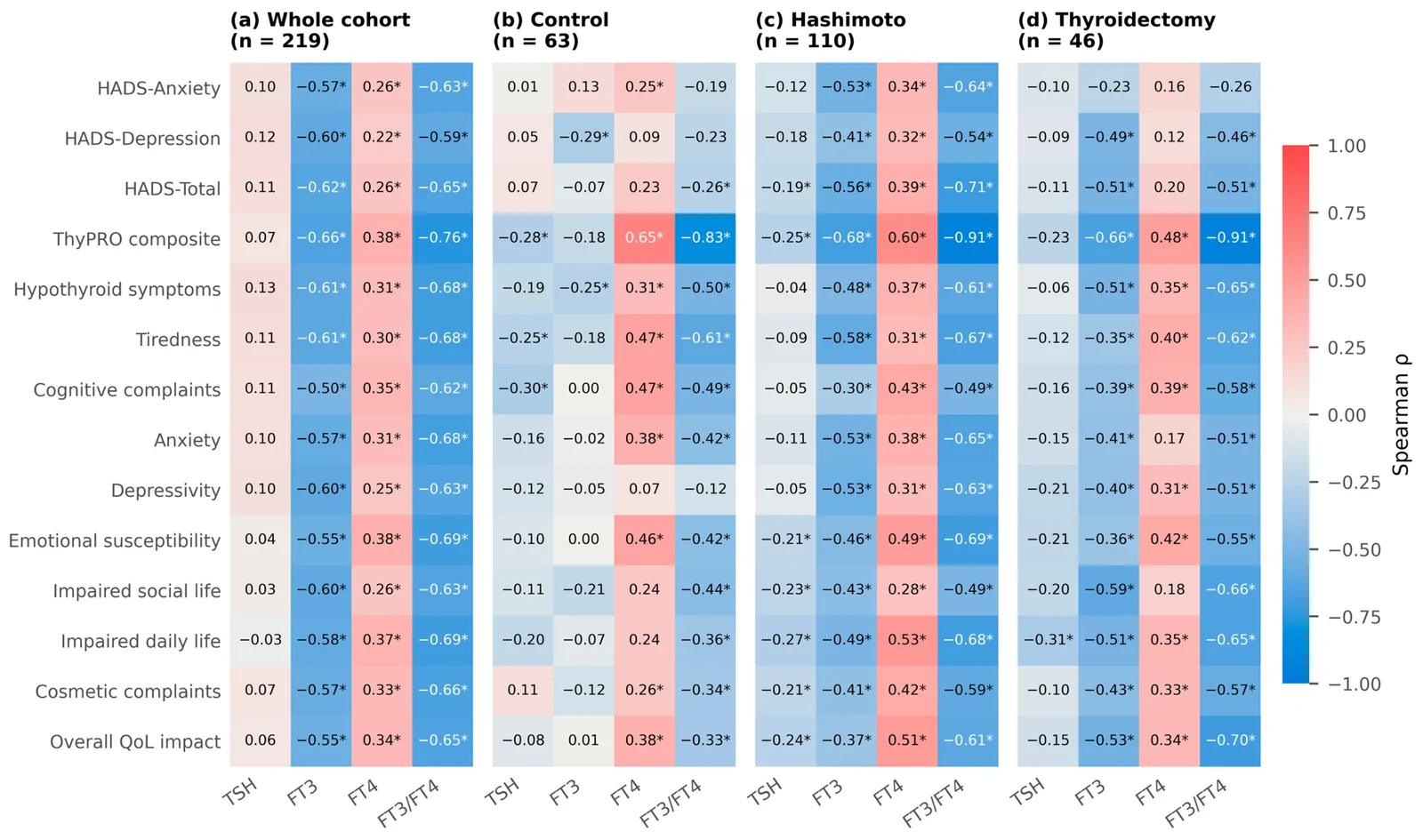
Spearman correlations of TSH, FT3, FT4 and the FT3/FT4 ratio with HADS and ThyPRO outcomes (**a**) in the whole cohort and within (**b**) controls, (**c**) Hashimoto patients and (**d**) thyroidectomy patients. Blue, inverse correlation; red, positive correlation; Exploratory analysis; * *p* < 0.05 (unadjusted). After Benjamini–Hochberg correction across all 224 coefficients, 144 of the 152 nominally significant correlations remained significant.

**Figure 4:**
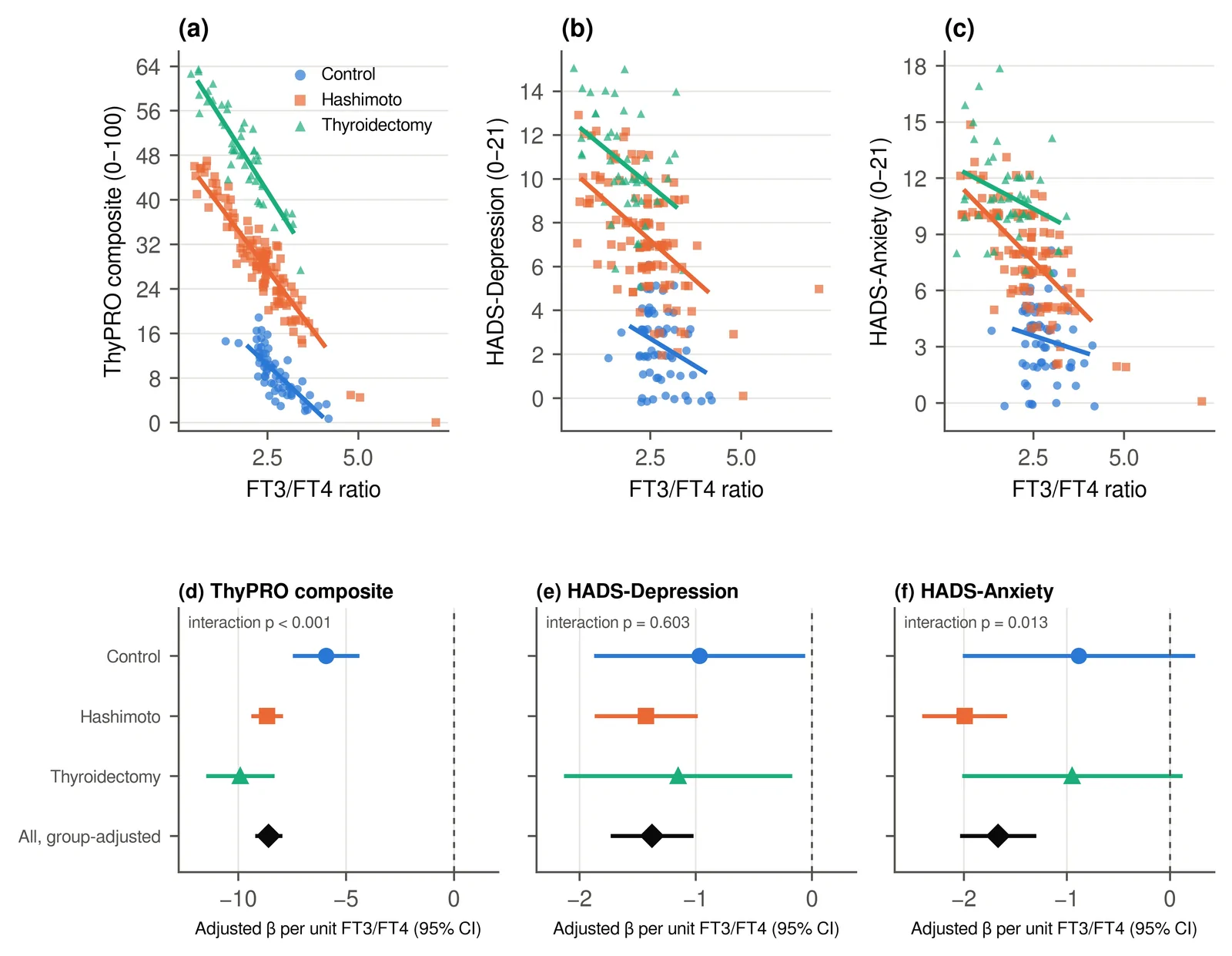
Association of the FT3/FT4 ratio with patient-reported outcomes. (**a**–**c**) ThyPRO composite, HADS-depression and HADS-anxiety scores against the FT3/FT4 ratio; lines are unadjusted least-squares fits within each group, drawn over the central 95% of that group’s ratio values. (**d**–**f**) Regression coefficients (β, 95% CI) per one-unit higher FT3/FT4 ratio within each group (adjusted for age, sex, BMI and TSH) and in all participants (additionally adjusted for group), with the *p*-value for the ratio × group interaction. Negative β indicates worse scores at lower ratios.

**Table 1:** Demographic, clinical and thyroid-function characteristics by group.

Characteristic	Control (*n* = 63)	Hashimoto (*n* = 110)	Thyroidectomy (*n* = 46)	*p*-Value
Age, years	43.2 ± 5.9	41.9 ± 6.2	40.8 ± 5.4	0.106
Female sex, n (%)	44 (69.8)	101 (91.8)	38 (82.6)	<0.001
BMI, kg/m^2^	26.5 ± 5.0	29.6 ± 6.1	30.0 ± 5.4	<0.001
Waist circumference, cm	85.2 ± 13.4	91.8 ± 15.2	94.8 ± 13.8	0.001
TSH, mIU/L	1.85 (1.22–2.29)	3.08 (1.60–4.35)	2.98 (1.21–4.94)	<0.001
FT3, pg/mL	3.30 (2.96–3.61)	2.83 (2.22–3.15)	2.53 (1.81–2.87)	<0.001
FT4, ng/dL	1.21 (1.08–1.37)	1.17 (1.05–1.35)	1.32 (1.15–1.61)	0.013
FT3/FT4 ratio	2.61 (2.41–3.05)	2.40 (1.78–2.85)	1.81 (1.34–2.24)	<0.001
TSH in range, n (%)	62 (98.4)	96 (87.3)	32 (69.6)	<0.001
TSH, FT3 and FT4 all in range, n (%)	50 (79.4)	63 (57.3)	11 (23.9)	<0.001

Data are mean ± SD, median (IQR) or n (%). *p*-values by one-way ANOVA (age, BMI, waist circumference), Kruskal–Wallis test (thyroid indices) or χ^2^ test. Reference ranges: TSH 0.33–5.33 mIU/L; FT3 2.13–4.50 pg/mL; FT4 0.58–1.38 ng/dL. The FT3/FT4 ratio is FT3 (pg/mL) divided by FT4 (ng/dL); multiply by 0.119 for the molar ratio. Lipids, other routine biochemistry and sex hormones are given in Table S1. BMI, body-mass index; FT3, free triiodothyronine; FT4, free thyroxine; TSH, thyrotropin.

**Table 2:** HADS and ThyPRO scores by group, with effect sizes.

Outcome	Control (*n* = 63)	Hashimoto (*n* = 110)	Thyroidectomy (*n* = 46)	η^2^	*p*-Value
HADS (0–21)					
HADS-Anxiety	4.0 (2.0–5.0)	8.0 (6.0–10.0)	11.0 (10.0–12.0)	0.564	<0.001
HADS-Depression	2.0 (1.0–4.0)	7.0 (6.0–9.0)	10.5 (9.0–12.0)	0.621	<0.001
HADS-Total	6.0 (4.0–7.5)	15.0 (13.0–19.0)	22.0 (19.0–23.8)	0.680	<0.001
ThyPRO (0–100)					
ThyPRO composite	8.4 (5.9–11.9)	28.7 (23.5–34.3)	48.9 (43.5–55.2)	0.753	<0.001
Hypothyroid symptoms	11.0 (3.0–17.0)	34.0 (26.3–44.8)	53.0 (45.0–62.8)	0.587	<0.001
Tiredness	10.0 (2.0–17.0)	37.0 (26.0–46.0)	64.5 (56.5–72.0)	0.670	<0.001
Cognitive complaints	8.0 (1.0–15.0)	28.0 (21.0–38.0)	54.5 (44.3–62.8)	0.645	<0.001
Anxiety	8.0 (1.0–14.0)	30.0 (20.0–39.8)	50.0 (43.3–56.8)	0.584	<0.001
Depressivity	8.0 (2.5–16.5)	32.0 (21.3–39.0)	51.0 (41.5–59.0)	0.595	<0.001
Emotional susceptibility	5.0 (0.0–13.0)	27.0 (17.0–38.0)	44.0 (40.3–56.0)	0.573	<0.001
Impaired social life	7.0 (0.0–14.5)	24.0 (17.0–31.0)	40.0 (30.3–49.5)	0.530	<0.001
Impaired daily life	5.0 (0.0–14.0)	26.0 (18.0–33.8)	43.0 (35.3–55.3)	0.518	<0.001
Cosmetic complaints	3.0 (0.0–10.5)	24.0 (14.0–29.8)	35.0 (27.0–44.8)	0.474	<0.001
Overall QoL impact	6.0 (0.0–14.0)	30.0 (20.5–38.8)	48.0 (42.3–61.8)	0.632	<0.001

Data are median (IQR); scores range 0–21 (HADS) or 0–100 (ThyPRO), with higher scores indicating worse status. The ThyPRO composite is the unweighted mean of the nine administered scales and the overall QoL-impact item. Between-group comparisons by Kruskal–Wallis test (η^2^, eta-squared effect size); all *p*-values remained <0.001 after Benjamini–Hochberg correction. Dunn post hoc tests with Bonferroni adjustment gave *p* < 0.001 for all three pairwise contrasts (control vs. Hashimoto, control vs. thyroidectomy and Hashimoto vs. thyroidectomy) for every outcome. HADS, Hospital Anxiety and Depression Scale; QoL, quality of life; ThyPRO, Thyroid-Related Patient-Reported Outcome.

**Table 3:** Association of the FT3/FT4 ratio with patient-reported outcomes: group-adjusted and group-specific models.

Outcome/Analysis	n	β (95% CI)	*p*-Value
ThyPRO composite (primary model R^2^ = 0.96; interaction *p* < 0.001)			
All participants, group-adjusted	219	−8.59 (−9.13 to −8.05)	<0.001
Control	63	−5.93 (−7.38 to −4.47)	<0.001
Hashimoto	110	−8.67 (−9.31 to −8.02)	<0.001
Thyroidectomy	46	−9.91 (−11.40 to −8.42)	<0.001
HADS-depression (primary model R^2^ = 0.72; interaction *p* = 0.603)			
All participants, group-adjusted	219	−1.38 (−1.71 to −1.04)	<0.001
Control	63	−0.97 (−1.86 to −0.08)	0.034
Hashimoto	110	−1.43 (−1.85 to −1.00)	<0.001
Thyroidectomy	46	−1.15 (−2.11 to −0.19)	0.020
HADS-anxiety (primary model R^2^ = 0.69; interaction *p* = 0.013)			
All participants, group-adjusted	219	−1.67 (−2.02 to −1.32)	<0.001
Control	63	−0.88 (−1.99 to 0.23)	0.116
Hashimoto	110	−1.99 (−2.39 to −1.60)	<0.001
Thyroidectomy	46	−0.95 (−2.00 to 0.10)	0.075

Linear regression. The all-participant model includes the FT3/FT4 ratio, diagnostic group, age, sex, BMI and TSH; group-specific models include the ratio, age, sex, BMI and TSH. β is the change in score per one-unit higher FT3/FT4 ratio (negative values indicate worse scores at lower ratios). Interaction *p*, F-test for the ratio × group interaction. The all-participant rows are the primary analysis (Holm-adjusted *p* < 0.001 for all three outcomes); group-specific estimates and interaction tests are exploratory. Robustness and sensitivity analyses are given in Table S4, and comparisons with models containing FT3 and FT4 in Table S5. BMI, body-mass index; CI, confidence interval; HADS, Hospital Anxiety and Depression Scale; TSH, thyrotropin.

**Table 4:** Exploratory discrimination of probable depression and anxiety by the FT3/FT4 ratio within patients.

Outcome	Cases, n (%)	AUC (95% CI)	Cut-Off	Sens./Spec., Apparent	Sens./Spec., Optimism-Corrected
Probable depression (HADS-D ≥ 8)	92 (59.0)	0.78 (0.70–0.85)	2.13	0.65/0.83	0.63/0.81
Probable depression (HADS-D ≥ 11)	37 (23.7)	0.79 (0.69–0.86)	1.86	0.76/0.77	0.72/0.77
Probable anxiety (HADS-A ≥ 8)	107 (68.6)	0.81 (0.75–0.87)	2.25	0.69/0.84	0.67/0.81
Probable anxiety (HADS-A ≥ 11)	41 (26.3)	0.77 (0.69–0.85)	2.18	0.80/0.65	0.77/0.64

ROC analysis within patients (Hashimoto + thyroidectomy, *n* = 156), with a lower FT3/FT4 ratio as the positive direction. AUC with 1000-sample bootstrap 95% CI. Cut-off by the Youden index; sensitivity and specificity are shown as apparent values and after bootstrap optimism correction (1000 resamples). Cut-offs refer to FT3 (pg/mL)/FT4 (ng/dL) on the assay used here and are exploratory. AUCs within each patient group and the comparison with a group-only model are given in Table S6. AUC, area under the receiver-operating-characteristic curve; HADS-A/-D, HADS anxiety/depression subscales.

## Data Availability

The data presented in this study are available on request from the corresponding author. The data are not publicly available due to privacy restrictions.

## Supplementary Materials

The following supporting information can be downloaded at: https://www.mdpi.com/article/10.3390/medicina62102017/s1, Figure S1: Participant flow. Consecutive eligible adults attending the endocrinology and internal-medicine outpatient clinics of Sivas Numune Hospital between 15 January and 15 July 2026 were invited. There were no missing data for any analysed variable. LT4, levothyroxine; Figure S2: Receiver-operating-characteristic curves of the FT3/FT4 ratio (lower values as the positive direction) for (a) probable depression and (b) probable anxiety within patients (Hashimoto + thyroidectomy, *n* = 156), at HADS cut-offs of ≥8 (solid) and ≥11 (dashed). Points mark the Youden-optimal cut-off. AUC with 1000-sample bootstrap 95% CI. The analysis is exploratory; cut-offs are specific to FT3 (pg/mL)/FT4 (ng/dL) on the assay used and have not been externally validated (see Table 4 of the main text for optimism-corrected sensitivity and specificity); Table S1: Routine biochemistry and sex hormones by group; Table S2: Probable depression and anxiety at HADS cut-offs of ≥8 and ≥11, and Firth penalised logistic regression within patients; Table S3: Spearman correlations (ρ, p) of thyroid indices with patient-reported outcomes, in the whole cohort and within each group, and of total testosterone with outcomes by sex; Table S4: Sensitivity and robustness analyses of the association of the FT3/FT4 ratio with patient-reported outcomes (β per one-unit higher ratio, 95% CI); Table S5: Comparison of linear models containing FT3, FT4 and the FT3/FT4 ratio (all participants, *n* = 219); Table S6: Discrimination of probable depression and anxiety by the FT3/FT4 ratio within each patient group, and comparison of group-only and group-plus-ratio logistic models (patients, *n* = 156).
