# Supplementary material for: Quality of Life and Mood in Relation to Free T3, Free T4 and the FT3/FT4 Ratio in Levothyroxine-Treated Hypothyroidism: A Cross-Sectional Study
Source: Medicina (Kaunas). 2026 Oct 18;62(10):2017. doi: 10.3390/medicina62102017 (PMC13706868; doi:10.3390/medicina62102017)
Supplement: Supplementary file 1 [file medicina-62-02017-s001.zip › medicina-4579401-supplementary.pdf]

Supplementary Materials

Quality of Life and Mood in Relation to Free T3, Free T4 and the FT3/FT4 Ratio in Levothyroxine-Treated Hypothyroidism: A Cross-Sectional Study

Gülşah Altun and Enver Çiftel

Contents: Figure S1 (participant flow); Figure S2 (ROC curves within patients); Table S1 (routine biochemistry and sex hormones); Table S2 (probable depression and anxiety at HADS cut-offs ≥ 8 and ≥ 11, and Firth logistic regression); Table S3 (within-group Spearman correlations); Table S4 (sensitivity and robustness analyses of the linear models); Table S5 (comparison of models containing FT3, FT4 and the FT3/FT4 ratio); Table S6 (ROC analysis within each patient group and comparison of group-only and group-plus-ratio models). All analyses other than the primary group-adjusted models are exploratory.

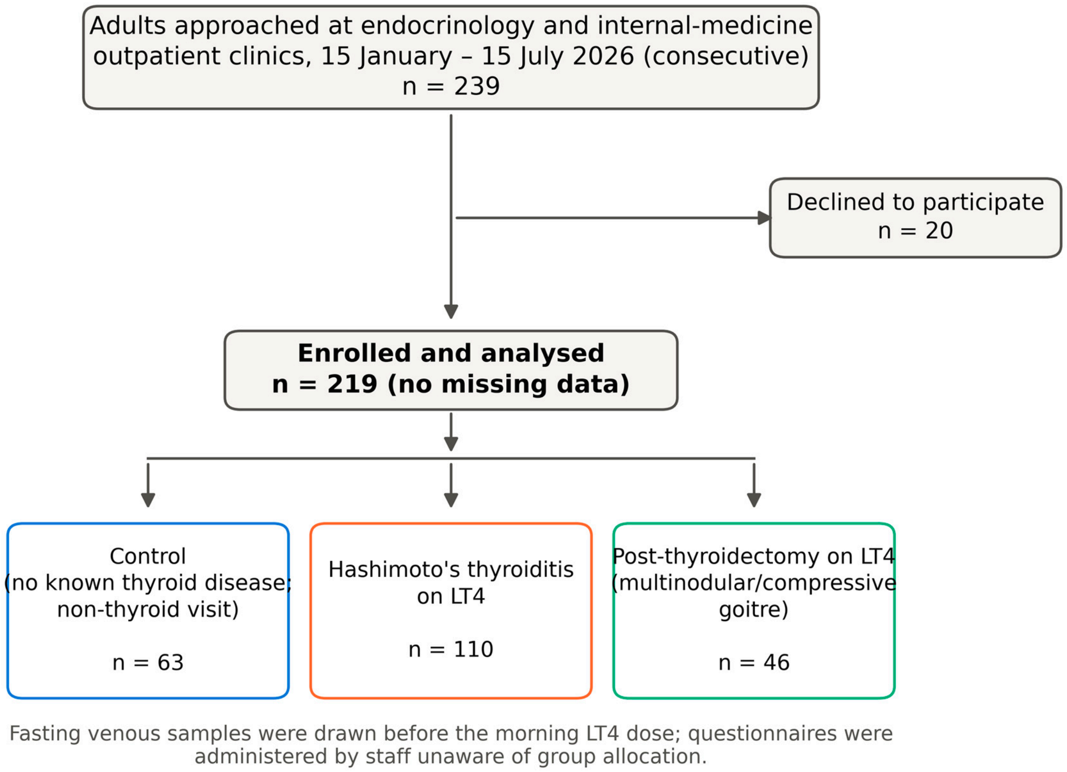

Figure S1. Participant flow. Consecutive eligible adults attending the endocrinology and internal-medicine outpatient clinics of Sivas Numune Hospital between 15 January and 15 July 2026 were invited. There were no missing data for any analysed variable. LT4, levothyroxine.

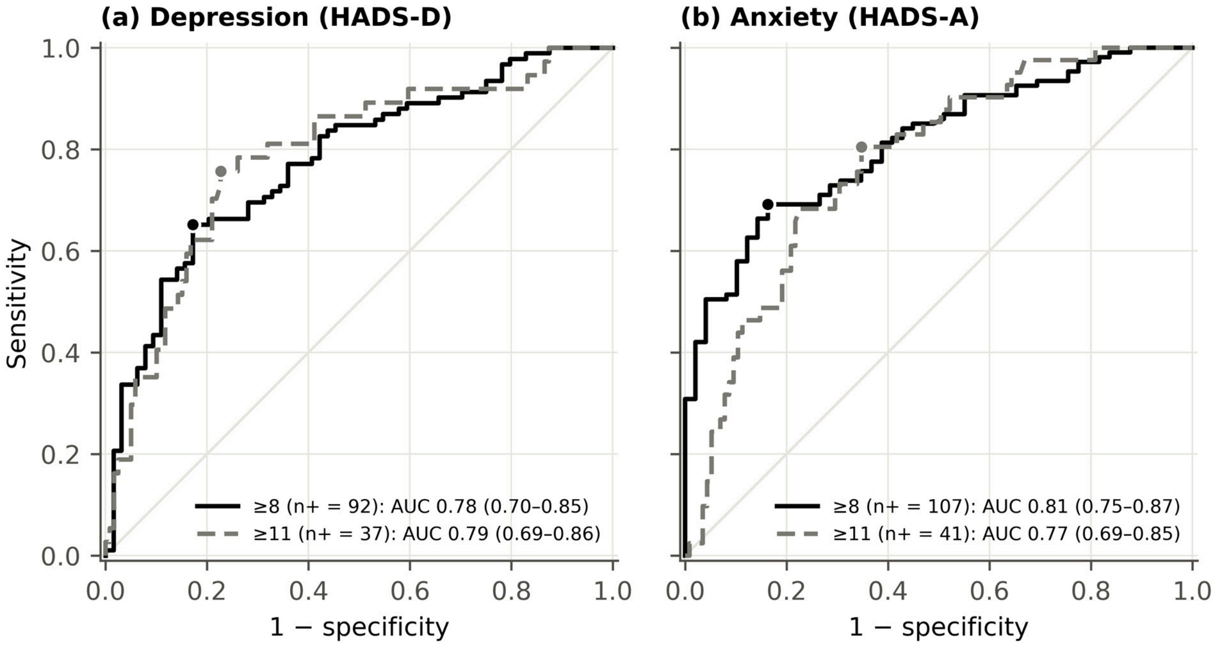

Figure S2. Receiver-operating-characteristic curves of the FT3/FT4 ratio (lower values as the positive direction) for (a) probable depression and (b) probable anxiety within patients (Hashimoto + thyroidectomy, $n = 156$), at HADS cut-offs of ≥ 8 (solid) and ≥ 11 (dashed). Points mark the Youden-optimal cut-off. AUC with 1000-sample bootstrap 95% CI. The analysis is exploratory; cut-offs are specific to FT3 (pg/mL)/FT4 (ng/dL) on the assay used and have not been externally validated (see Table 4 of the main text for optimism-corrected sensitivity and specificity).

Table S1. Routine biochemistry and sex hormones by group.

Variable	Control (n = 63)	Hashimoto (n = 110)	Thyroidectomy (n = 46)	p-Value
Fasting glucose, mg/dL	87.0 (79.0–93.0)	92.0 (87.0–103.8)	91.0 (85.0–100.0)	<0.001
Total cholesterol, mg/dL	175.0 (151.0–205.0)	192.0 (162.0–228.5)	210.5 (183.2–231.8)	0.003
LDL-C, mg/dL	117.0 (88.0–138.0)	124.5 (103.0–156.8)	139.0 (114.2–159.8)	0.004
HDL-C, mg/dL	52.0 (40.5–59.5)	49.5 (42.0–57.0)	48.5 (41.2–64.8)	0.689
Triglycerides, mg/dL	97.0 (60.0–152.5)	115.0 (84.2–162.2)	127.0 (82.8–178.8)	0.014
Uric acid, mg/dL	4.70 (4.00–5.60)	4.90 (4.00–5.60)	4.90 (4.00–5.85)	0.840
ALT, U/L	17.0 (11.0–25.5)	16.0 (12.2–22.8)	15.0 (12.2–19.8)	0.644
AST, U/L	17.0 (14.0–21.5)	18.0 (15.0–20.0)	17.0 (16.0–20.8)	0.886
GGT, U/L	15.0 (11.0–23.5)	15.0 (11.0–20.0)	17.0 (11.0–23.0)	0.367
Total bilirubin, mg/dL	0.45 (0.34–0.64)	0.43 (0.33–0.59)	0.50 (0.34–0.66)	0.435
Direct bilirubin, mg/dL	0.20 (0.10–0.25)	0.20 (0.10–0.20)	0.20 (0.10–0.20)	0.301
Creatinine, mg/dL	0.72 (0.66–0.86)	0.73 (0.67–0.81)	0.70 (0.63–0.90)	0.999
Haemoglobin, g/dL	14.0 (12.9–15.2)	13.8 (12.7–14.6)	13.6 (13.1–14.4)	0.303
Platelets, $\times10^3/\mu L$	270.0 (231.0–306.5)	297.5 (257.2–340.8)	250.0 (222.5–298.0)	<0.001
Neutrophils, $\times10^3/\mu L$	3.97 (3.02–4.91)	4.15 (3.25–5.19)	3.44 (2.69–4.52)	0.034
Lymphocytes, $\times10^3/\mu L$	2.14 (1.81–2.60)	2.13 (1.80–2.52)	2.18 (1.77–2.48)	0.743
**Sex hormones, women (Control n = 44; Hashimoto n = 101; Thyroidectomy n = 38)**				
Total testosterone, ng/dL	36.5 (27.8–47.0)	32.0 (24.0–43.0)	35.5 (26.0–47.0)	0.500
SHBG, nmol/L	48.0 (33.5–70.5)	42.0 (29.0–59.0)	44.5 (31.0–59.8)	0.299
FSH, mIU/mL	5.68 (3.76–7.06)	10.00 (5.80–48.00)	36.45 (5.56–62.95)	<0.001
LH, mIU/mL	7.3 (5.0–11.4)	15.4 (7.0–31.0)	20.2 (8.5–36.0)	<0.001
Oestradiol, pg/mL	68.0 (39.8–123.0)	42.0 (13.0–121.0)	18.5 (2.0–68.9)	0.002
**Sex hormones, men (Control n = 19; Hashimoto n = 9; Thyroidectomy n = 8)**				
Total testosterone, ng/dL	419.0 (340.5–524.5)	411.0 (328.0–536.0)	313.0 (260.8–393.2)	0.174
SHBG, nmol/L	29.0 (23.5–39.0)	30.0 (24.0–44.0)	25.0 (20.8–31.8)	0.659
FSH, mIU/mL	4.30 (2.55–6.59)	4.00 (2.75–7.16)	3.87 (2.90–4.22)	0.686
LH, mIU/mL	5.00 (4.13–7.64)	4.10 (3.64–6.67)	5.99 (3.94–7.37)	0.748
Oestradiol, pg/mL	16.2 (9.6–22.8)	22.3 (17.0–30.3)	21.2 (10.6–33.1)	0.257

Data are median (IQR); p-values by Kruskal–Wallis test. Sex hormones are reported separately for women and men. Units as reported by the laboratory. ALT, alanine aminotransferase; AST, aspartate aminotransferase; FSH, follicle-stimulating hormone; GGT, gamma-glutamyl transferase; HDL-C/LDL-C, high-/low-density lipoprotein cholesterol; LH, luteinising hormone; SHBG, sex-hormone-binding globulin.

Table S2. Probable depression and anxiety at HADS cut-offs of ≥ 8 and ≥ 11, and Firth penalised logistic regression within patients.

Outcome	Control (n = 63)	Hashimoto (n = 110)	Thyroidectomy (n = 46)	χ^2 p	Firth OR (95% CI)	p
Depression, HADS-D ≥ 8	0 (0.0)	51 (46.4)	41 (89.1)	<0.001	0.32 (0.18–0.53)	<0.001
Depression, HADS-D ≥ 11	0 (0.0)	14 (12.7)	23 (50.0)	<0.001	0.30 (0.15–0.55)	<0.001
Anxiety, HADS-A ≥ 8	2 (3.2)	63 (57.3)	44 (95.7)	<0.001	0.19 (0.08–0.37)	<0.001
Anxiety, HADS-A ≥ 11	0 (0.0)	16 (14.5)	25 (54.3)	<0.001	0.30 (0.16–0.52)	<0.001

Prevalence shown as n (%); χ^2 test across the three groups. The cut-off of ≥ 8 was prespecified; ≥ 11 (the Turkish validation cut-off for anxiety) was added as a sensitivity analysis during revision. Firth models within patients (n = 156), adjusted for group, age, sex, BMI and TSH; odds ratio per one-unit higher FT3/FT4 ratio, with profile-likelihood 95% CI and likelihood-ratio p. Controls were excluded from the logistic models because none met the depression thresholds (complete separation). OR, odds ratio.

Table S3. Spearman correlations (ϱ, p) of thyroid indices with patient-reported outcomes, in the whole cohort and within each group, and of total testosterone with outcomes by sex.

Outcome	TSH	FT3	FT4	FT3/FT4 ratio
**Whole cohort (*n* = 219)**				
ThyPRO composite	0.07 (0.275)	−0.66 (<0.001)	0.38 (<0.001)	−0.76 (<0.001)
HADS-depression	0.12 (0.071)	−0.60 (<0.001)	0.22 (0.001)	−0.59 (<0.001)
HADS-anxiety	0.10 (0.146)	−0.57 (<0.001)	0.26 (<0.001)	−0.63 (<0.001)
HADS-total	0.11 (0.104)	−0.62 (<0.001)	0.26 (<0.001)	−0.65 (<0.001)
**Control (*n* = 63)**				
ThyPRO composite	−0.28 (0.027)	−0.18 (0.152)	0.65 (<0.001)	−0.83 (<0.001)
HADS-depression	0.05 (0.670)	−0.29 (0.020)	0.09 (0.468)	−0.23 (0.067)
HADS-anxiety	0.01 (0.929)	0.13 (0.321)	0.25 (0.047)	−0.19 (0.142)
HADS-total	0.07 (0.572)	−0.07 (0.598)	0.23 (0.067)	−0.26 (0.042)
**Hashimoto (*n* = 110)**				
ThyPRO composite	−0.25 (0.008)	−0.68 (<0.001)	0.60 (<0.001)	−0.91 (<0.001)
HADS-depression	−0.18 (0.060)	−0.41 (<0.001)	0.32 (<0.001)	−0.54 (<0.001)
HADS-anxiety	−0.12 (0.211)	−0.53 (<0.001)	0.34 (<0.001)	−0.64 (<0.001)
HADS-total	−0.19 (0.042)	−0.56 (<0.001)	0.39 (<0.001)	−0.71 (<0.001)
**Thyroidectomy (*n* = 46)**				
ThyPRO composite	−0.23 (0.122)	−0.66 (<0.001)	0.48 (<0.001)	−0.91 (<0.001)
HADS-depression	−0.09 (0.551)	−0.49 (<0.001)	0.12 (0.443)	−0.46 (0.001)
HADS-anxiety	−0.10 (0.515)	−0.23 (0.118)	0.16 (0.299)	−0.26 (0.083)
HADS-total	−0.11 (0.473)	−0.51 (<0.001)	0.20 (0.190)	−0.51 (<0.001)

Values are Spearman ϱ (unadjusted p).

Total testosterone vs.	ThyPRO composite	HADS-depression	HADS-anxiety
Women (*n* = 183)	0.00 (0.998)	−0.02 (0.808)	−0.01 (0.883)
Men (*n* = 36)	−0.26 (0.121)	−0.27 (0.117)	−0.33 (0.049)

Spearman ϱ (p) between serum total testosterone and each outcome, by sex. HADS, Hospital Anxiety and Depression Scale.

Table S4. Sensitivity and robustness analyses of the association of the FT3/FT4 ratio with patient-reported outcomes (β per one-unit higher ratio, 95% CI).

Analysis	n	ThyPRO composite	HADS-depression	HADS-anxiety
**Robustness of the primary model (all participants, group-adjusted)**				
Primary model (OLS)	219	−8.59 (−9.13 to −8.05)	−1.38 (−1.71 to −1.04)	−1.67 (−2.02 to −1.32)
OLS with HC3 robust standard errors	219	−8.59 (−9.71 to −7.47)	−1.38 (−1.86 to −0.89)	−1.67 (−1.99 to −1.34)
Huber robust regression	219	−9.03 (−9.58 to −8.47)	−1.43 (−1.78 to −1.07)	−1.71 (−2.06 to −1.37)
Excluding influential observations (Cook's D > 4/n)	206–211	−9.19 (−9.73 to −8.65) [n = 211]	−1.52 (−1.88 to −1.15) [n = 210]	−1.79 (−2.10 to −1.47) [n = 206]
Excluding the extreme ratio value (7.14)	218	−9.10 (−9.66 to −8.54)	−1.55 (−1.91 to −1.18)	−1.68 (−2.07 to −1.30)
FT3 instead of the ratio (β per 1 pg/mL FT3)	219	−7.03 (−8.11 to −5.95)	−1.39 (−1.78 to −1.00)	−1.34 (−1.77 to −0.90)
**Subgroups (same covariates; group term omitted where only one group)**				
TSH within reference range	190	−8.98 (−9.56 to −8.40)	−1.41 (−1.81 to −1.02)	−1.75 (−2.17 to −1.33)
TSH, FT3 and FT4 all within reference range	124	−7.75 (−8.81 to −6.68)	−1.59 (−2.37 to −0.82)	−1.40 (−2.16 to −0.63)
Women only	183	−8.71 (−9.28 to −8.14)	−1.38 (−1.75 to −1.00)	−1.63 (−2.01 to −1.25)
Patients only (Hashimoto + thyroidectomy)	156	−8.93 (−9.52 to −8.34)	−1.41 (−1.79 to −1.03)	−1.79 (−2.17 to −1.41)

Analysis	n	ThyPRO composite	HADS-depression	HADS-anxiety
Excluding the 13 controls with FT4 above the reference range	206	−8.66 (−9.21 to −8.12)	−1.43 (−1.77 to −1.08)	−1.69 (−2.05 to −1.33)

All models adjusted for diagnostic group, age, sex, BMI and TSH (sex omitted in the women-only model); the FT3 row replaces the ratio with FT3. All $p < 0.001$. Subgroup sizes: TSH in range, control 62, Hashimoto 96, thyroidectomy 32; all three tests in range, 50, 63 and 11. Diagnostics of the primary models: Shapiro–Wilk p for residuals 0.21, 0.81 and 0.91; Breusch–Pagan $p <$ 0.001, 0.054 and 0.71; variance inflation factor for the ratio 1.23. OLS, ordinary least squares; HC3, heteroscedasticity-consistent standard errors.

Table S5. Comparison of linear models containing FT3, FT4 and the FT3/FT4 ratio (all participants, n = 219).

Model	R^2	Partial R^2 of added terms	AIC	BIC	CV RMSE
**ThyPRO composite**					
Base (group, age, sex, BMI, TSH)	0.780	—	1518.4	1542.1	7.77
Base + FT3	0.876	0.438	1394.1	1421.2	5.86
Base + FT4	0.846	0.301	1442.1	1469.2	6.56
Base + FT3 + FT4	0.944	0.743	1224.7	1255.2	4.06
Base + log FT3 + log FT4	0.953	0.787	1183.8	1214.3	3.65
Base + FT3/FT4 ratio	0.961	0.824	1139.9	1167.0	3.32
Base + log FT3/FT4 ratio	0.952	0.781	1188.2	1215.3	3.68
Base + FT3 + FT4 + ratio	0.965	0.840	1123.7	1157.6 *	3.23 *
**HADS-depression**					
Base (group, age, sex, BMI, TSH)	0.635	—	992.7	1016.4	2.35
Base + FT3	0.704	0.189	948.7	975.9	2.12
Base + FT4	0.649	0.038	986.1	1013.2	2.31
Base + FT3 + FT4	0.718	0.229	939.8	970.3	2.08
Base + log FT3 + log FT4	0.720	0.234	938.3	968.8	2.07 *
Base + FT3/FT4 ratio	0.720	0.233	936.6	963.7 *	2.07
Base + log FT3/FT4 ratio	0.717	0.227	938.5	965.6	2.07
Base + FT3 + FT4 + ratio	0.730	0.261	932.4	966.3	2.07
**HADS-anxiety**					
Base (group, age, sex, BMI, TSH)	0.562	—	1025.9	1049.7	2.52
Base + FT3	0.628	0.150	992.3	1019.5	2.34
Base + FT4	0.597	0.081	1009.5	1036.6	2.43
Base + FT3 + FT4	0.664	0.232	972.1	1002.6	2.24
Base + log FT3 + log FT4	0.668	0.242	969.3	999.8	2.23
Base + FT3/FT4 ratio	0.691	0.295	951.5	978.6 *	2.13 *
Base + log FT3/FT4 ratio	0.667	0.240	967.7	994.8	2.21
Base + FT3 + FT4 + ratio	0.693	0.300	953.9	987.8	2.14

All models include diagnostic group, age, sex, BMI and TSH. Partial R^2, proportion of residual variance of the base model explained by the added term(s). CV RMSE, root-mean-square prediction error from 10-fold cross-validation repeated 10 times (lower is better). * lowest value for that outcome. Nested F-tests: the ratio added to FT3 ($p < 0.001$, < 0.001, < 0.001 for ThyPRO composite, HADS-depression and HADS-anxiety) and to FT3 and FT4 together ($p < 0.001$, 0.003, < 0.001); FT3 added to the ratio ($p < 0.001$, 0.006, 0.363). Test of equal and opposite coefficients for log FT3 and log FT4 (the constraint implied by a log ratio): p 0.014, 0.154, 0.520. Exploratory analysis; AIC, Akaike and BIC, Bayesian information criterion.

Table S6. Discrimination of probable depression and anxiety by the FT3/FT4 ratio within each patient group, and comparison of group-only and group-plus-ratio logistic models (patients, n = 156).

Outcome	AUC, Hashimoto (n = 110)	AUC, thyroidectomy (n = 46)	AUC, group only	AUC, group + ratio	ΔAUC (95% CI)	CV AUC, group / group + ratio
Depression, HADS-D	0.76 (0.67–0.85)	NE (41/46)	0.68	0.82	0.14 (0.09–	0.59 / 0.81

Outcome	AUC, Hashimoto (*n* = 110)	AUC, thyroidectomy (*n* = 46)	AUC, group only	AUC, group + ratio	ΔAUC (95% CI)	CV AUC, group / group + ratio
≥ 8					0.20)	
Depression, HADS-D ≥ 11	0.82 (0.71–0.90)	0.75 (0.59–0.89)	0.71	0.84	0.12 (0.08–0.20)	0.62 / 0.81
Anxiety, HADS-A ≥ 8	0.78 (0.68–0.86)	NE (44/46)	0.69	0.85	0.17 (0.11–0.22)	0.59 / 0.84
Anxiety, HADS-A ≥ 11	0.82 (0.69–0.92)	0.61 (0.43–0.78)	0.71	0.83	0.12 (0.06–0.20)	0.62 / 0.81

Within-group AUCs with 1000-sample percentile bootstrap 95% CI, lower ratio as the positive direction. NE, not estimated because fewer than 10 patients were in one outcome class (cases/n shown): at the ≥ 8 cut-offs only 5 (depression) and 2 (anxiety) thyroidectomy patients were below the threshold. Group-only and group-plus-ratio models are logistic regressions in all patients; ΔAUC with 1000-sample bootstrap 95% CI; likelihood-ratio test for adding the ratio $p < 0.001$ for all four outcomes. CV AUC, 10-fold cross-validated AUC, repeated 10 times. Exploratory analysis.
